# Operation of a triage committee for advanced life support during the COVID-19 pandemic

**DOI:** 10.1186/s13010-022-00117-1

**Published:** 2022-03-16

**Authors:** Benjamín Herreros, Rafael Ruiz de Luna, Natalia de la Calle, Diego Gayoso, Paula Martínez, Karmele Olaciregui Dague, Gregorio Palacios

**Affiliations:** 1 Internal Medicine Unit, Alcorcón Foundation University Hospital, Alcorcón, Spain; 2 Francisco Vallés Institute of Clinical Ethics, European University, Madrid, Spain; 3 Intensive Medicine Unit, Alcorcón Foundation University Hospital, Madrid, Spain; 4 Epileptology Department, University Hospital Bonn, Bonn, Germany

**Keywords:** COVID-19, Decision-making, Ethics Committees/Consultation, Triage

## Abstract

**Background:**

During the first weeks of March 2020 in Spain, the cases of severe respiratory failure progressively increased, generating an imbalance between the clinical needs for advanced life support (ALS) measures and the effective availability of ALS resources. To address this problem, the creation of triage committees (TC) was proposed, whose main function is to select the best candidates to receive ALS. The main objective of our study is to describe the clinical characteristics of the patients evaluated by the TC of the Alcorcón Foundation University Hospital (AFUH) during the first wave of SARS CoV-2. Other objectives are to determine if there are differences between the patients considered candidates / not candidates for ALS and to analyze the functioning of the TC.

**Methods:**

Retrospective observational study of all patients assessed by the AFUH TC.

**Results:**

There were 19 meetings, in which 181 patients were evaluated, 65.4% male and with a mean age of 70.1 years. 31% had some degree of functional dependence, the Barthel median was 100 and Charlson 4. 58.5% were not considered a candidate for ALS at that time. The patients considered candidates to receive ALS were younger (72 vs 66; *p* < 0.001), had less comorbidity (Charlson 4 vs 3; *p* < 0.001) and had a better previous functional situation. A median of 5 physicians participated in each meeting and, after being assessed by the TC, 13.6% received ALS: 29.3% of those considered candidates for ALS and 2% of the non-candidates.

**Conclusions:**

The patients evaluated by the TC had a mean age of 70 years, high comorbidity and almost a third had some degree of functional dependence. More than half were not considered candidates for ALS at that time, these patients being older, with more comorbidity and a worse previous functional situation. TC decisions, based on objective clinical criteria, were almost always respected. Public institutions must get involved in triage procedures, which should and in our opinion must include the creation of TC in health centers. The implementation of Anticipated Decision programs (ADP) would help enable patients affected by triage decisions to participate in them.

## Background

In March 2020, the number of cases with Severe Acute Respiratory Syndrome (SARS) CoV-2 in Spain grew alarmingly, generating an overload of the healthcare system. COVID-19 fulfilled the characteristics foreseen as a health disaster [1]: a situation in which the destructive effects of an event exceed the capacity of an area or community to meet the healthcare needs of its inhabitants. During the first weeks of March 2020, previous estimates pointed to an increase in the number of cases of severe respiratory failure in the following weeks, which threatened to generate an imbalance between the clinical needs for advanced life support measures (ALS) and the effective availability of ALS resources.

Triage processes are responsible for assessing and classifying patients to determine the priority of their care and the most appropriate location for their treatment [2]. To triage is to ensure that each patient, based on available resources, receives the best treatment in the shortest time possible. We carry out different types of triage in our healthcare system: in transplants, in emergencies, with some especially expensive therapies (for example, in vitro fertilization), and so on. However, with respect to beds in the Intensive Care Units (ICU) and ALS, before the pandemic in Spain triage was not performed. Many patients did not enter the ICU, though not due to of a lack of respirators, but because it was considered that it would not benefit them to enter the ICU. These decisions are not about triage, but about limitation of life support (withdrawal or withholding of life support treatment decisions), which consist of not applying life support to patients with a poor prognosis and poor quality of life, because applying it would be futile or disproportionate, that is, it would cause them a damage proportionally greater than the potential benefit [3].

During the first wave of the SARS CoV-2 pandemic, triage had to be carried out to access the ALS in many neighbouring countries [4, 5]. But it cannot be ignored that, compared to other developed countries, the Spanish health system was not well prepared to face a pandemic. The *Global Health Security Index* [6] compares the response capacity of countries to a pandemic based on their preventive capacity, the resources of the health system and the capacity to treat patients. Within Europe, in 2019 Spain ranked 15th (65.9 points out of 100 possible), ranking among the countries with average preparation. Regarding the beds available in the event of a potential health disaster, the EU had 5.1 hospital beds for every 1,000 people, whereas Spain had 3.0, ranking 24th in the UE-28 [7]. Considering the five most populous countries in Europe, Spain was only ahead of the United Kingdom: Germany had 8.1 beds per 1,000 people, France 6.1, Italy 3.1, and the United Kingdom 2.6. Regarding intensive care beds [8], though these data are difficult to obtain reliably [9], Spain was far from France or Germany, a country that almost tripled Spain’s endowment of intensive care beds per inhabitant [10, 11].

However, although the Spanish healthcare system was not among the best prepared in Europe to face the pandemic, an enormous effort was made to increase the beds and resources of intensive care. Despite this, in certain areas there was an imbalance between the needs and the available resources of ALS. The similarity of our reality with that of other international experiences reported (China, Italy), made it unacceptable not to establish recommendations that would help prioritize care during periods of shortage of ALS resources. If there is an imbalance between the needs and the available resources of ALS, it is necessary to have criteria and procedures that help make triage decisions in a rational and fair way. In March 2020, it was an ethical obligation to establish procedures and criteria of distributive justice so that triage decisions were not random.

A proposed procedure to make triage decisions rationally and based on objective criteria is to create Triage Committees (TC) [12]. The purpose of these committees is to carry out a comprehensive evaluation of patients who are candidates for ALS, considering the available resources, in order to select the best candidates for ALS. In addition, TCs relieve the healthcare team of the moral and emotional burden of triage decisions [13]. Health centers where triage decisions have to be made should establish a TC that would operate permanently while such decisions have to be made. In this way, decisions would be made by an independent group of professionals based on previously established objective criteria. It has been recommended that these committees be made up of intensive care specialists, attending physicians who care for COVID-19 patients, and members of the Clinical Ethics Committee who are clinicians and who ideally treat patients with COVID-19. All of them must be familiar with the ethical principles that guide triage and the clinical criteria on which triage decisions should be based.

During the second week of March 2020, some hospitals in Madrid (one of the regions where the spread of the pandemic was most intense) began to run out of ALS resources. In the absence of national or regional guidelines to perform triage in the best possible way, health centers were forced to establish their own criteria to prioritize care: the Infanta Elena University Hospital (Department of Clinical Bioethics) did so first on March 10, followed on March 13 by the Hospital Universitario La Princesa (Clinical Ethics Interconsultation Service), and on 16 March by the Hospital Universitario Fundación Alcorcón, and later by Hospital Clínico San Carlos, Hospital La Paz, etc.

At the Alcorcón Foundation University Hospital (AFUH), a Triage Committee (TC) was set up in order to make triage decisions on ALS in a rational way and according to criteria of distributive justice. The TC was intended to help clinicians make decisions on an ethical basis: basing them on objective clinical criteria applicable to all possible candidates [13] (Table 1). It was also conceived to guarantee that clinical practice was of the highest quality according to available resources, to reduce uncertainty in decision-making and to support clinicians, relieving them of part of the responsibility, as triage decisions are very difficult and emotionally taxing [14].

**Table 1:** Clinical criteria for the decisions of the HUFA Triage Committee

PREVIOUS SITUATION: •Ten year life expectancy according to age and comorbidities (modified Charlson Comorbidity index) •Functional status and previous quality of life of the patient (Barthel index for functional assessment)
CURRENT SITUATION: •Probability of survival (recovery) of the acute process based on the current clinical situation (severity / gravity). The APACHEII Scale, validated for use 24 h after admission to the ICU, serves as a guideline
•In extreme situations, in similar cases (due to life expectancy and functional situation), age will be considered, giving priority to patients with more potential years of life saved

The main objective of the article is to describe the clinical characteristics of patients evaluated by the AFUH TC during the first wave of SARS CoV-2. Other objectives are to determine if there are differences between the patients considered candidates / not candidates for ALS and to analyze the functioning of the TC.

## Methods

### Study design

The present is a retrospective observational study, through the review of the clinical history (CH) and the daily registry of the TC, of all patients evaluated by the TC of the AFUH. The TC evaluated all potential candidates for ALS, even if at that time they were stable and did not require ALS. In this way, it was established in advance whether, in case of clinical worsening, a patient was a candidate to receive ALS. The TC did not assess patients who, due to their clinical characteristics, would not benefit from receiving ALS (as this measure was futile or disproportionate).

### Operation of the triage committee

Every day the hospital's COVID-19 team provided the TC with a list of patients who could require ALS, specifying whether they were currently stable or could require ALS in the next hours / days. The TC met at 12:00 p.m. First, the TC determined the ALS resources immediately available, as well as the resources that could be made available through patient transfer. After that, the list of patients was analyzed, to determine (based on their clinical characteristics) which patients were the best candidates to receive ALS at that time. The role of TC was, therefore, to determine which patients could benefit the most from ALS. The TC functioned as an advisory committee, interpreting the criteria for triage according to the specific situation of the hospital: needs and available resources of ALS. Daily deliberation and decisions were recorded in a log.

After each meeting, the physicians involved (ward clinicians and intensivists) were informed of which unstable patients were candidates for ALS at that time and which stable patients could become candidates in case of clinical worsening, so that they could organize their care and communication tasks in the best way possible. In some cases, patients were classified as candidates (or not candidates) to receive ALS at that time, with obligatory reevaluation if they worsened. The decisions of the TC were recorded in the HC.

### Variables

Following variables were recorded for each patient: age, gender, meeting (or meetings) in which they were assessed, functional grade according to the Barthel index, comorbidities according to the modified Charlson index. Certain comorbidities that are usually undervalued by the Charlson index were also recorded: morbid and premorbid obesity (grade 3–4), severe lung disease (OSAHS, COPD, PHT), severe heart disease (valvular heart disease, ischemic heart disease, cardiomyopathy), degree of cognitive impairment (according to the Clinical Dementia Rating; CDR), congenital intellectual disability (DSM-5 defines intellectual disabilities as neurodevelopmental disorders that begin in childhood and are characterized by intellectual difficulties as well as difficulties in conceptual, social, and practical areas of living) and disabling psychiatric disorder (severe psychiatric illness that prevents the realization of an autonomous life). The outcome of the TC evaluation was also collected (the patient is a candidate for ALS; they are not a candidate for ALS; they are a candidate at that time but must be reevaluated if they worsen; they are not a candidate at that time but should be reevaluated if they worsen), whether the patient eventually received ALS, and whether the patient had died one month after the TC evaluation.

To analyze the functioning of the TC, the number of professionals who participated in the daily deliberation of the TC and their affiliation was recorded: ICU, Anesthesiology, Internal Healthcare Ethics Committee and COVID team. The number of patients assessed at each meeting and the decision made in each case was also recorded.

### Statistical analysis

The data were analyzed using SPSS Statistics 17® (IBM, Armonk, NY, USA). Qualitative variables were described using absolute and relative frequencies and quantitative variables with mean and standard deviation (SD) or median and interquartile range (IQR: p25-p75), depending on the distribution of the data. An exploratory univariate analysis was carried out to study possible differences in patients evaluated by the TC according to the outcome, using the chi-square test for qualitative variables and Student's t-test or the Mann–Whitney U test, according to the distribution of the data, in the case of quantitative variables. The Wilson method was used to estimate the 95% confidence interval (95% CI) of a proportion. The level of statistical significance was established at an alpha error of *p* < 0.05.

### Compliance with ethical standards

The study was carried out in compliance with the principles and ethical standards of the Declaration of Helsinki (revised version by Forteza, 2013), the Oviedo Convention of the Council of Europe (1997) and the Good Clinical Practices of the International Conference on Harmonization (GCP of the ICH, 1996). The study was also approved by the AFUH Research Ethics Committee.

We collected data electronically, including sensitive data, in accordance with the legislation on personal data in force in Spain, Organic Law 3/2018, of December 5, on Protection of Personal Data and guarantee of digital rights, and in the EU, Regulation (EU) 2016/679 of the European Parliament and of the Council of April 27, 2016 on Data Protection.

## Results

The TC had the first meeting on 03/19/2020 and the last on 04/14/2020, the day that, for the first time since TC implementation, no problems were detected with available ALS resources. In total, there were 19 meetings, in which 181 patients were evaluated. 65.4% were male and the mean age was 70.1 years (SD: 9.7). 31% had some degree of functional dependence, the median Barthel index was 100 (IQR: 90–100) and the median modified Charlson index was 4 (IQR: 3–5), which indicates a high comorbidity. 6.1% of the patients assessed had morbid / premorbid obesity, 19.5% severe lung disease, 3.4% severe heart disease, 4.4% cognitive impairment, 5.5% congenital intellectual disability and 1.7% disabling psychiatric disorder.

More than half of patients (58.5% (95% CI: 51.3% -65.5%) were considered not a candidate for ALS at the time. However, 6.6% were deemed as needing reassessment in case of clinical worsening. Regarding those considered candidates to receive ALS at the time (41.4%), 6.1% had to be reevaluated if they worsened (Table 2).

**Table 2:** Decisions made by the Triage Committee

**Decision**	Total	*Not candidate for ICU*	*Candidate for ICU*	*Not a candidate, but must be reevaluated*	*Candidate, but must be reevaluated*
Nº patients	181	94 (51,9%)	64 (34,4%)	12 (6,6%)	11 (6,1%)
Age	70,1 ± 9,7	72,66 ± 8,69	66,88 ± 10,47	72,92 ± 4,1	64,18 ± 11,11
Charlson Index	4 (3–5)	4 (3–6)	3 (3—4)	3,5 (3–4)	3 (3–5)
Barthel Index	100 (90–100)	100 (85–100)	100 (100–100)	100 (65–100)	100 (70–100)

*N* (%), Mean ± standard deviation, Median (interquartile range)

The patients considered candidates to receive ALS were younger (72.69 ± 8.28 vs 66.48 ± 10.53; *p* < 0.001), they had less comorbidity (median [IQR]: 4 [3–6] vs 3 [3-4]; *p* < 0.001) and better functional situation (median [IQR]: 100 [85–100] vs 100 [98–100]; *p* = 0.053). 25% of the subjects who were candidates for ALS had a Barthel lower than 95, a figure that drops to 85 in those not candidates for ALS. Of the patients with moderate-severe functional dependence, 83% did not receive ALS. This was also the case in 56% of functionally independent patients. Regarding the patients with cognitive impairment, 87% (7 of the 8 assessed) did not receive ALS. Table 3 shows the characteristics of the patients considered candidates / non-candidates for ALS.

**Table 3:** Characteristics of the patients evaluated by the Triage Committee

Variable	ICU candidate?		*p*-value
	**No**	**Yes**	
	*n* = 106	*n* = 75	
**Sex**			
Male	68 (57,1%)	51 (42,9%)	0,591
Female	38 (61,3%)	24 (38,7%)	
**Age**			
Median ± Deviation	72,69 ± 8,28	66,48 ± 10,53	< 0,001
**Charlson Index**			
Median (p25-p75)	4 (3—6)	3 (3—4)	< 0,001
**Barthel Index**			
Median (p25-p75)	100 (85—100)	100 (98—100)	0,053
**Cognitive Impairment**			
No	98 (57%)	74 (43%)	0,242
Slight	6 (85,7%)	1 (14,3%)	
Moderate	1 (100%)		
**Dementia according to Charlson Index**			
No	100 (57,5%)	74 (42,5%)	0,242
Yes	6 (85,7%)	1 (14,3%)	
**Functional dependency**			
Moderate-Severe	10 (83,3%)	2 (16,7%)	0,197
Slight	24 (60%)	16 (40%)	
Independent	65 (56,5%)	50 (43,5%)	
**Functional dependency**			
Dependence	34 (65,4%)	18 (34,6%)	0,280
Independence	65 (56,5%)	50 (43,5%)	
**Congenital intellectual disability**			
No	100 (59,2%)	69 (40,8%)	0,325
Yes	4 (40%)	6 (60%)	
**Disabling psychiatric disorder**			
No	101 (57,4%)	75 (42,6%)	0,266
Yes	3 (100%)		
**Obesity 3–4**			
No	97 (58,1%)	70 (41,9%)	1,000
Yes	7 (63,6%)	4 (36,4%)	
**Severe pulmonary disease**			
No	82 (56,9%)	62 (43,1%)	0,525
Yes	22 (62,9%)	13 (37,1%)	
**Severe cardiopathy**			
No	99 (57,6%)	73 (42,4%)	0,403
Yes	5 (83,3%)	1 (16,7%)	

A median of 5 physicians participated in each meeting (IQR: 5–6; 2 from ICU, 1 from Anesthesia and Resuscitation, 1 from the COVID team and 2 from the Internal Healthcare Ethics Committee). A median of 9 patients (IQR: 7–12) were evaluated at each meeting and a median of 3 (IQR: 2–4) were considered candidates for ALS. After being assessed by TC, 13.6% (*n* = 24, 95% CI: 9.3% -19.5%) received ALS. 91.6% of them (22 of the 24 who received ALS) had initially been considered a candidate to receive ALS, with 29.3% of the ALS candidates receiving ALS and 2% of the non-candidates. One month after being assessed by the TC, 19.2% (95% CI: 14% -25.7%) of the patients had died, 14.9% of the candidates and 22.4% of the not candidates.

## Discussion

The AFUH TC was intended to help clinicians make ethically based triage decisions, basing them on objective clinical criteria. In the 19 meetings of the TC, 181 patients were evaluated, with a mean age of 70 years, high comorbidity and almost a third with some degree of functional dependence. More than half of the patients were considered not candidates for ALS at the time, these patients were older (72 vs 66) and with more comorbidity (Charlson median 4 vs 3), reaching statistical significance in these two parameters. Although the Barthel index is at the limit of statistical significance, patients who were not candidates for ALS had a worse functional situation. With regard to functional status and quality of life, those with moderate-severe dependency did not usually receive ALS, and the majority of patients with cognitive impairment (7 out of 8) were considered not candidates for ALS.

For triage decisions to be rational and justified, they must be based on ethical principles (in this case, seeking the greatest benefit for the greatest number of patients) and on objective clinical criteria [15]. Did the TC fulfill its mission to help make decisions based on objective clinical criteria? The answer must be affirmative, because decisions responded to the predefined clinical criteria: life expectancy based on age and comorbidities, functional status / quality of life, and probability of recovery from the acute process, which led the candidates to ALS were younger, had less comorbidity, better functional status, less functional dependence and cognitive impairment.

In situations of triage for ALS, in addition to defining recommendations and criteria, it is advisable that there be a committee to help make decisions [16, 17]. TCs must be interdisciplinary, including physicians from the departments involved in decisions (intensive care, emergency and ward doctors) and specialists in bioethics [18]. These TCs, in addition to helping to interpret the criteria for triage decisions (making clinical practice, relative to resources, of the highest quality), can reduce the uncertainty inherent in triage, because decisions are made by a set of qualified professionals [19]. Finally, it should not be forgotten that TCs are a support for healthcare workers, who display moral distress and are sometimes overwhelmed by having to make these types of decisions [20, 21]. The objective of medicine is the optimal care of the health of patients [22] and, if there are not enough resources, this care cannot be given to all those who require it. Performing triage has serious emotional consequences for professionals, who can be overwhelmed and demoralized by a situation for which they are not always prepared [23, 24]. If a committee helps them make these decisions, clinicians are relieved of some of the responsibility, mitigating the psychological discomfort and moral injury that accompanies triage decisions [25].

The meetings were regularly attended by 5 doctors from the ICU, Anesthesia and Resuscitation, the COVID team and the Internal Healthcare Ethics Committee. TC decisions were almost always taken into account: 29.3% of the patients considered candidates for ALS eventually received ALS, whereas only 2% of the non-candidates did so. If triage decisions are made by an independent group of professionals and respond to objective criteria and arguments, the healthcare professionals responsible for the patients better understand the decision. Furthermore, their actions will be consistent with decisions that they feel are justified, no matter how difficult and emotionally taxing it may be.

One of the main problems detected in triage decisions about ALS and in TCs is that patients affected by the decisions and their relatives hardly participate in decision-making. Decisions are made, in general, by health professionals and there is hardly any opinion from those affected. As far as possible, patient views should be incorporated into decision-making, desirably in advance. For this reason, it would be very useful to develop Advance Care Planning (ACP) programs, especially in patients with advanced cardiovascular and respiratory disease [26], who are more likely to be affected by triage decisions. An ACP program of health decisions is the result of a process of reflection and relationship-building between the patient, their relatives and health professionals. It is based on respect for patients’ autonomy, involving them in making decisions about their illness in a way that is shared between the medical team, the patient and their relatives. In an ACP program all the possible future scenarios must be explain to the patient and patient’s values must be known by the healthcare team and entered into their medical record, so as to ensure the patients’ wishes are respected if there is a situation in which they are unable to express their care preferences. There are evidences that ACP programs are useful for decision-making and, besides, these programs can improve patient-related outcomes such as patient satisfaction with care, quality of communication and shared decision-making [27]. ACP programs for decision-making have been carried out in Spain in diseases like chronic kidney disease [28, 29], or in advance heart failure [26]. The characteristics of patients with severe respiratory failure who may need ALS make it an appropriate model for designing ACP programs, given the high prevalence of these patients during the pandemic, with a progressive increase in its incidence, and because of it’s clinical characteristics: a low life expectancy without ALS and the ability to foresee future clinical scenarios on which is possible to plan decision-making [30]. However, until now ACP programs have not been put into practice in patients affected by severe respiratory failure for whom triage decisions are necessary. What is this due to? Maybe because ACP programs have not been considered, or because of the difficulty of put them into practice in this scenario, since are serious patients at high risk of losing the ability to make decisions in a very short time. Nevertheless, ACP programs are developed precisely to planning decisions for patients who are at risk of losing the ability to decide. To do this in the context of triage decisions on ALS, patients have to be adequately informed by the healthcare team with the support of the TC, so that patients can establish their preferences. Possible future clinical scenarios must be foreseen with the patients so that they can express their wishes. And, finally, the preferences and wishes of the patients must be taken into account by the TC in its deliberations. Hence, in the face of possible triage decisions on ALS, ACP programs should be promoted with patients candidate to triage, so that, as far as possible, the decisions of the patients are respected.

This study has the limitations that retrospective works usually display. Regarding its strengths, it should be noted that it is the first study to shed light on the functioning and usefulness of a TC. The study opens up numerous questions that must be answered in the future: who should be part of these committees, whether the healthcare workers responsible for the patients should participate in the deliberation, whether the decisions should be binding, or whether the patient and their relatives should be informed of said decisions.

## Conclusions

Our main conclusions are the following: the patients evaluated by the AFUH TC during the first wave of SARS CoV-2 had a mean age of 70 years, high comorbidity and almost a third had some degree of functional dependence. More than half were not considered a candidate for ALS at the time, these patients being older, with more comorbidity and a worse previous functional situation. Usually 5 physicians participated in TC deliberations and their decisions, based on objective clinical criteria, were almost always respected. The situation of imbalance between the needs and resources of ALS experienced in March 2020 should not happen again. But if it is repeated, public institutions must get involved and define the criteria and procedure to carry out triage, which should and in our opinion must include the creation of TC in health centers [31]. Leaving all this responsibility to healthcare workers is a departure from the governance obligations of health authorities. Lastly, the implementation of ACP programs would help enable patients affected by triage decisions to participate in them.

## Data Availability

The datasets used and/or analysed during the current study available from the corresponding author on reasonable request.

## Abbreviations

TC: Triage committee
ALS: Advanced life support
AFUH: Alcorcón Foundation University Hospital
ACP: Advance Care Planning
